# Supplementary material for: Developing a youth-friendly internet-enabled HIV risk calculator: A collaborative approach with young key populations, living in Soweto, South Africa
Source: PLOS Digit Health. 2025 Jan 31;4(1):e0000672. doi: 10.1371/journal.pdig.0000672 (PMC11785273; doi:10.1371/journal.pdig.0000672)
Supplement: S1 File — (DOCX) [file pdig.0000672.s001.docx]

INTERVIEW GUIDE FOR IN-DEPTH INTERVIEWS

Thank you for agreeing to be part of this in-depth interview. In the next hour we will be talking about your experience in HIV testing and counselling and how we can develop a youth friendly HIV Risk Calculator. We will look at the HIV Risk Assessment Questionnaire and discuss its content and how we can make it appropriate and user friendly for young people. We will also talk about how the Risk Assessment questionnaire can be adapted into an internet-based HIV Risk Calculator. Kindly complete the questionnaire on demographics and risk assessment.

HIV testing Experience

1. Have you ever tested for HIV before? What was the experience like? Did you talk about your risks for HIV with the counsellor? How did you feel about that?
2. How honest do you think you were when responding to the HIV counsellor about your sexual behaviour?
3. How comfortable did you think you were to talk about your sexual experiences and behaviour?
4. How do you think your responses would be different if you were completing a risk assessment in your private space on your cell phone?
5. Given the nature of your work (as sex worker), what challenges do you think you still experience in accessing HIV testing services? (only ask sex workers)
6. Given the nature of your sexual orientation (as Lesbian/Gay/Bisexual), what challenges do you think you still experience in accessing HIV testing services?

Risk Assessment Questionnaire

1. What did you think about the questions on the Risk Assessment questionnaire?
2. How do you think the questions are relevant for young people living in Soweto?
3. What questions did you find irrelevant to assess risk for young people living in Soweto?
4. What risk factors do you think are missing from the Risk Assessment questionnaire?
5. What do you think are the reasons young people test for HIV?
6. How do you think the questions are easy to understand for young people?
7. How do you think the Risk Assessment Questionnaire can be developed youth friendly and easily accessible for young people living in Soweto?
8. What do you think young people use their cell phones for when on the internet?
9. What do you know about MyMsta cell phone application designed by Lovelife?
10. Have you ever searched for HIV information on the internet or through your cell phone? If yes, what prompted you to do so? If no, why?
11. What kind of HIV information were you searching for?
12. If we develop an HIV Risk Calculator accessible through cell phone or other internet gadgets, what do you think would be the barriers for young people living in Soweto to use it?
13. How can the HIV Risk Calculator be most effectively used by (young LGBs/female sex workers) through cell phones and internet access?
