# Supplementary material for: Developing a youth-friendly internet-enabled HIV risk calculator: A collaborative approach with young key populations, living in Soweto, South Africa
Source: PLOS Digit Health. 2025 Jan 31;4(1):e0000672. doi: 10.1371/journal.pdig.0000672 (PMC11785273; doi:10.1371/journal.pdig.0000672)
Supplement: Attachment — Submitted filename: RebuttalLetter-PDIG-D-23-00013-R1-05-DEC-23.pdf [file pdig.0000672.s004.pdf]

ASSURE
African Social Sciences Unit of Research and Evaluation

12th Floor, New Nurses Home
Chris Hani Baragwanath Academic Hospital
Diepkloof
1864
+27 11 989 9831

Rebuttal letter for manuscript: PDIG-D-23-00013R1

Title: Developing a youth-friendly internet-enabled HIV risk calculator: A collaborative approach with young people including key population aged 18–24 years living in Soweto, South Africa".

Dear Editor,

Thank you very much for the review of our manuscript and for affording us the opportunity to respond to reviewers comments. Below is our response to the comments in blue text.

Journal comment:
Thank you for providing an Ethics Statement for your study. Currently, your Ethics Statement appears outside of the Methods section of your manuscript (page 2). Please ensure that the Ethics Statement appears only in your Methods section and remove any other occurrences.

Response
The authors have rechecked the manuscript and found that the ethics statement only appears in the methods section of the manuscript under the subheahing 'Ethical considerations', on pages 13-14.

Reviewer 2 comments:

Comment 1
The authors have thoroughly addressed the reviewer comments, which has helped with clarity. I suggest reviewing for minor edits, such as to make sure that all acronyms are defined at first use (for example, the acronyms PGDs and IDIs are used on page 9 of the tracked changes version under "Study population and setting", but not defined until later in the manuscript).

Response:
Thank you for the comment, the acronyms PGDs and IDIs are spelt in full. Peer Group Discussions for PGDs and In-Depth Interviews for IDIs. See page seven paragraph one.

Comment 2
There are also some participant quotations that are repeated in both Table 3 and in the main text; these could be removed from one of the locations to reduce repetition.

Response:

The following quotation was identified as repeating in Table 3 and the main text and was therefore removed from Table 3. *"I think this is a very good start but then you also need to understand that young people are visual people and if it's going to be an app, it has to be very visual and it has to be something that attracts them. They get quite easily bored; just try not to make something so monotone. The reason why the Instagram, the Twitters and everything take on so wonderfully is that it's very graphic. You are able to follow people's pictures, looking at people's pictures[be]cause we want to see, you want that visual stimulation."* ***(IDI011, female, 24 years old)".***

Comment 3
Finally, when reporting participant demographics (e.g., in Table 1 and in the subsection titled "Participant demographics and mobile phone usage"), I recommend using the terms "cisgender male" and "cisgender female" (as the counterparts to "transgender" or "transgender female") rather than "male" and "female" alone.

Response

The terms male and female were changed to cisgender male and cisgender female in table 1 and in the subsection titled 'Participant demographics and mobile phone usage' on page 14 of the manuscript.

Regards,

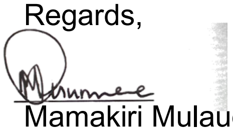

Mamakiri Mulaudzi

African Social Sciences Unit of Research and Evaluation (ASSURE) is a research unit and operates as a division of the Wits Health Consortium

The Wits Health Consortium (Pty) Limited (WHC) is wholly-owned by the University of the Witwatersrand.
(Pty) Ltd. Company Registration No: 97/15443/07

Executive Directors: Prof Janan Dietrich and Dr Nellie Myburgh
