# Supplementary material for: Developing a youth-friendly internet-enabled HIV risk calculator: A collaborative approach with young key populations, living in Soweto, South Africa
Source: PLOS Digit Health. 2025 Jan 31;4(1):e0000672. doi: 10.1371/journal.pdig.0000672 (PMC11785273; doi:10.1371/journal.pdig.0000672)
Supplement: Attachment — Submitted filename: RebuttalLetter-PDIG-D-23-00013-R4-17-SEP-2024.pdf [file pdig.0000672.s008.pdf]

ASSURE
African Social Sciences Unit of Research and Evaluation

12th Floor, New Nurses Home
Chris Hani Baragwanath Academic Hospital
Diepkloof
1864
+27 11 989 9831

Rebuttal letter for manuscript: PDIG-D-23-00013R3 **17 September 2024**

Title: Developing a youth-friendly internet-enabled HIV risk calculator: A collaborative approach with young people including key population aged 18–24 years living in Soweto, South Africa".

Dear PLOS Digital Health Journal Editor,

Thank you very much for the manuscript review and for affording us the opportunity to respond to the reviewers' comments. Kindly see our line by line response to the comments.

Response to Reviewer # 5

Comment 1: Line 29: Would consider avoid using colloquial/informal terms such as "tap"
Response 1: Thank you for your feedback. We have revised and rephrased the sentence by replacing the term 'tap' with 'leverage'. The sentence now reads as follows: "Concerns about confidentiality impede the utilization of HIV prevention services, which signals the need for discrete HIV prevention measures that leverage on youth-friendly platforms".

Comment 2: HIV risk calculator (HRC): if the editor agrees, I would avoid using the acronym HRC as it is not commonly used, and thus difficult to understand for readers that may have skipped a section.
Response 2: We agree with the reviewer. We have removed the acronym "HRC" and use the full name "HIV risk calculator" throughout the document.

Comment 3: Typo/grammar line 31: unnecessary "the"
Response 3: The sentence has been revised to read as follows: "This paper describes the process of developing a youth-friendly internet-enabled HIV risk calculator in collaboration with young people, including young key populations aged between 18 and 24 years old". See page 2, line 28-30.

Comment 4: "young key populations groups" please correct to "young key population groups"
Response 4: Thank you for your observation. We made the correction as suggested. The phrase "young key populations groups" has been corrected to "young key population groups". See page 2, line 37-38.

Comment 5: Line 34 to 38: Very long sentence, consider splitting

Response 5: Th e sentence was concised as suggested. See page 2, line 34-39. The sentence now reads as follows: Eligible participants were young people aged between 18–24 years old and living in Soweto. Data was collected through two peer group discussions with young people aged 18-24 years, a once-off group discussion with the [Name of clinic removed for confidentiality] adolescent community advisory board members and once off face-to-face in-depth interviews with young key population groups: lesbian, gay, bisexual, transgender (LGBT) individuals, men who have sex with men (MSM), and female sex workers.

Comment 6: Line 40: sentence structure confusing

Response 6: The sentence was revised, see page 2 line 42-44. The sentence reads as follows: The measures used to collect data included a socio-demographic questionnaire, a questionnaire on mobile phone usage, an HIV and STI risk assessment questionnaire, and a semi-structured interview guide.

Comment 7: Consider outlining in abstract why the selected groups are “key populations”, I assume because they are groups at highest risk of HIV infection, or rather because they are less likely to access HIV care?

Response 7: Yes, we agree with the reviewer and we added a sentence to clarify in the abstract why the groups are considered “key populations.” The sentence added on page 2, line 39-42 reads as follows: “LGBT individuals are identified as key populations because they face increased vulnerability to HIV/AIDS and other health risks due to societal stigma, discrimination, and obstacles in accessing healthcare and support services”.

Comment 8: Conclusion: “Privacy, confidentiality, and ease of use promote acceptability and willingness to use internet-enabled HIV prevention methods.” Should be reworded to be less assertive. Consider using terms such as “Participants found/emphasized/highlighted” etc.

Response 8: Thank you for the observation. The concluding sentence ws rephrased as suggested. The sentence on page 3, line 56-57 reads as follows: “Participants emphasized that privacy, confidentiality, and ease of use contribute to the acceptability and willingness to use internet-enabled HIV prevention methods”.

Comment 9: Line 60: “Facilities are available” reword

Response 9: As suggested the sentence was rephrased for readability. See page 3, line 63-66, the sentence reads as follows: “There are facilities available for HIV testing, treatment and management however, barriers to confidentiality prevail, including long queues at testing facilities, and lack of youth-specific health care services, which is jeopardizing young people’s futures and lives”.

Comment 10: Line 61: Not clear why “long gueues at testing facilities and lack of youth-specific health care services” are barriers to confidentiality. Please explain or modify sentence

Response 10: The sentence about long ques at testing facilities and lack of youth-specific health care services was removed. See page 3, line 63-65.

Comment 11: Lack of confidentiality jeopardizes young people's futures and lives. Is a big statement. Is this what was meant?
Response 11: The comment was addressed by reframing the statement to clarify the potential consequences of lacking confidentiality for young people. The revised response specifically outlines how barriers like long queues and the absence of youth-specific health care services can discourage young individuals from seeking essential medical treatment, thereby risking untreated health issues and impacting their overall well-being.

Comment 12: No mention about how the HIV risk calculator helps youth assess their risk of HIV infection (is this not the main goal?).
Response 12: thank you for this observation. We added a paragraph to describe the HIV risk calculator and how it may be used to self assess the risk of HIV infection. See page 13, line 269-275, the following paragraph was added: "The aim of the HIV risk calculator is to provide an opportunity for young people to assess their own risk for HIV infections by anonymously answering a series of questions about sexual practices, preferences and possible exposure to HIV infection. Based on the responses given by the user, the HIV risk calculator will generate a report with information about the possible type of risk identified and what the user can do to reduce their risk for HIV infection. The HIV risk calculator is self-administered and anonymous".

Comment 13: Line 78: consider rewording
Response 13: The sentence was reworded. See page 4, line 78-79, and the sentence reads as follows: Among individuals aged 15-24 years, an estimated 56,000 new HIV infections were reported in 2022.

Comment 14: Line 91: "serious worry" consider rewording
Response 14: The phrase was changed from "serious worry" to "concern,". See page 5, line 109.

Comment 15: Line 141 typo
Response 15: We could not find the typo, however, we believe that the typo may have been corrected in one of the revisions rounds.

Comment 16: Line 169: "They could contact the study team as a walk-in" not clear what that means.
Response 16: The sentence was changed from " they could contact the study team as a walk-in" to "They could contact the study team either by visiting in person at the clinic…" See page 8, line 173-174.

Comment 17: Line 209 typo
Response 17: the sentence was changed and now reads as follows: Before the discussion, IDI participants completed a paper-based self-administered, short five-minute socio-demographic questionnaire, a questionnaire on mobile phone usage, and a 10-minute paper-based HIV and STI risk assessment questionnaire.

Comment 18: Line 215:" IDIs concluded with a discussion around the HRC" what was discussed "around the HRC"?

Response 18: The following sentence was added on page 10-11, line 220-222: "IDIs concluded with a discussion around the aestehtics and design of the HIV risk calculator" (see interview guide 2, under supplementary documents, for details on the type of questions asked)

Comment 19: Line 286: Would suggest providing equivalent amount in dollars or euros in parenthesis beside R150, for reader's outside of South Africa to understand.

Response 19: on page 14, line 306, we added the equivalent amount in US dollars. The sentence reads as follows: Participants were reimbursed R150 ($8.25) to cover the costs of transport and refreshments.

Comment 20: The HIV risk calculator was evaluated with young South Africans as part of a PhD degree and results are available in the form of a thesis on the database of the University of the Witwatersrand in Johannesburg South Africa. "Would remove this.

Response 20: Thank you for the suggestion and we removed the information.

Comment 21: There are many grammatical and language structure errors in some sections of the manuscript (abstract in particular) that would need to be corrected before it is ready to publish. Some were highlighted in this review, but many were not.

Response 21: This comment was addressed by thoroughly reviewing and correcting the manuscript for grammar, language structure, and coherence. Specific errors highlighted in the preceding comments were corrected, and additional proofreading was conducted to ensure the entire manuscript meets publication standards.

Comment 22: More details would be required to understand the IDI "purposive sampling" approach. From the way it reads, it sounds like a convenience sample using a chain referral strategy including participants who met the study's inclusion criteria

Response 22: This was addressed by providing a detailed explanation of the IDI purposive sampling approach. This included specifying the inclusion criteria, outlining the active community recruitment methods, and describing the change sampling, which clarified the distinction from a convenience sample.

Comment 23: After completing the HRC prototype" Does this mean a third round of PGD was held? Please clarify including the necessary information to understand the modality of this PGD, or reword to clarify what this means.

Response 23: Only a series of 2 PGDs were conducted in this study. The original sentence was rephrased to read as follows: "During the second PGD participants were asked to explore their preferences for mobile phone-based apps". See page 10, line 206-207.

Comment 24: At no point in the manuscript does it outline how the HIV risk calculator works, in the spirit of the principles for digital development, and for other groups to better understand the tool, it would be important to share how the calculator works

Response 24: The following paragraph was added to provide information about how the HIV risk calculator works. "The aim of the HIV risk calculator is to provide an opportunity for young people to assess their own risk for HIV infections by anonymously answering a series of questions about sexual practices, preferences and possible exposure to HIV infection. Based on the responses given by the user, the HIV risk calculator will generate a report with information about the possible type of risk identified and what the user can do to reduce their risk for HIV infection. The HIV risk calculator is self-administered and anonymous". See page 13, line 269-275.

Comment 25: The discussion could benefit from a mention on the impact of the modifications made to the digital app through the collaborative development process. Potential for improved uptake? Improved accuracy of the risk calculator?

Response 25: The following sentence was added to address the comment. The modifications made to the HIV risk assessment questions and the App through the collaborative process may present the opportunity for improved accuracy of the HIV risk assessment and acceptability of the App among young people in South Africa.see page 34, line 544-546

Comment 26: A framework such as the principles for digital development should be mentioned to highlight what aspects were followed, and highlight which aspects were not followed as limitations

Response 26:The following paragraphs were added in the discussion and limitation sections, respectively.

Our study applied the Principles for Digital Development framework[37], focusing on four of the nine principles. The nine principles being: design with the user; understand the existing ecosystem; design for scale; build for sustainability; be data driven; use open standards, open data, open source and open innovation; reuse and improve; address privacy and security and be collaborative[37]. By applying the four principles of digital development, being: design with the user; reuse and improve; address privacy and security and be collaborative, our study was able to collaboratively develop an HIV risk calculator that is youth-friendly, acceptable, confidential, discreet and user interface design. See page 30, line 452-459

The study was a small-scale research project and therefore could not apply some of the principles outlined in the Prinsiples for Digital Development which included: understanding the existing ecosystem; design for scale; build for sustainability; be data driven; use open standards, open data, open source and open innovation; reuse and improve. This was mainly due to the limited scope of the research project and therefore further studies can be conducted to improve the uptake, accessibility of the HIV risk calculator and influence policy in HIV prevention efforts in South Africa. See page 35, line 557-563

Additional changes

We removed the acronym HTS and replaced with the full text "HIV testing services". See page 10, line 217

We added the keyword "HIV risk calculator" at the section on keywords. See page 4, line 70-71

Thank you for the opportunity to revise our manuscript for possible publication in your journal. A very big thank you to the reviewers for the valuable feedback on the manuscript. We hope that the revisions made will be to your satisfaction.

Sincerely,

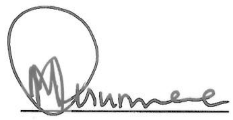

Dr Mamakiri Mulaudzi
https://orcid.org/0000-0001-8495-2209
tel:+27119899831/011 717 8331
https://assureafrica.com/
mamakirim@assureafrica.com / mamakiri.mulaudzi@wits.ac.za

African Social Sciences Unit of Research and Evaluation (ASSURE) is a research unit and operates as a division of the Wits Health Consortium

The Wits Health Consortium (Pty) Limited (WHC) is wholly-owned by the University of the Witwatersrand. (Pty) Ltd. Company Registration No: 97/15443/07

Executive Directors: Prof Janan Dietrich and Dr Nellie Myburgh
