# Supplementary material for: Developing a youth-friendly internet-enabled HIV risk calculator: A collaborative approach with young key populations, living in Soweto, South Africa
Source: PLOS Digit Health. 2025 Jan 31;4(1):e0000672. doi: 10.1371/journal.pdig.0000672 (PMC11785273; doi:10.1371/journal.pdig.0000672)
Supplement: Attachment — Submitted filename: PDIG-D-23-00013_Reviewers-Comments_20Aug2023.docx [file pdig.0000672.s003.docx]

PDIG-D-23-00013
Developing a youth-friendly internet-enabled HIV risk calculator: A collaborative approach with young people aged 18–24 years living in Soweto, South Africa
PLOS Digital Health

| Reviewer #1: |  |
| --- | --- |
| comments | Response |
| Have the authors made all data underlying the findings in their manuscript fully available (please refer to the Data Availability Statement at the start of the manuscript PDF file)?<br><br> The <a href="<https://journals.plos.org/digitalhealth/s/data-availability>" target="_blank">PLOS Data policy</a> requires authors to make all data underlying the findings described in their manuscript fully available without restriction, with rare exception. The data should be provided as part of the manuscript or its supporting information, or deposited to a public repository. For example, in addition to summary statistics, the data points behind means, medians and variance measures should be available. If there are restrictions on publicly sharing data—e.g. participant privacy or use of data from a third party—those must be specified.</font> | Data availability statement updated to “In accordance with the provisions of the Protection of Personal Information Act 4 of 2013, participants must consent for their data to be shared with third parties. However, at the time of study approval and data collection, participants had not consented for their data to be shared with third parties.  therefore, to maintain participant privacy and confidentiality, the qualitative datasets generated and/or analysed during the current study are not publicly available. The data would be accessible through email correspondence to [Mamakirim@assureafrica.com](mailto:Mamakirim@assureafrica.com) |
| Thank you for this paper, which describes inviting young people to pilot test and comment on an app to assess HIV risk prior to finalising the development of the app. Although an interesting read, the paper does not add sufficiently to the available evidence on co-development or mHealth; at times, the paper also lacks clarity on what the app would achieve  (ie. How would accurate risk of assessment overcome barriers to accessing available services; at times the app seems to focus only HIV and other times all STIs) | The HIV risk calculator is aimed to provide young people, young key population with a tool to self-assess for HIV risk. Based on the responses that participants give to risk assessment questions, the HIV risk calculator will generate a report to explain why certain sexual bahaviour activities are considered high risk. The tool also provide information on where to receive free HIV and counselling and testing services. |
| why different populations of young people were asked to comment differently on the app. | The research project had 2 phased. The PGDs formed phase one of the study which focused on developing and designing the initial prototype of the HIV risk calculator. Phase one of the study also focused on reviewing the HIV and STI assessment questionnaire. The PGDs recruited young people irrespective of gender, sexual identity and practices. Gay, lesbian, bisexual and MSM also were identified in the PGDs even though they were fewer than in the IDIs.  The IDIs formed phase two of the research project which included young people and especially targeted to include key population. The IDIs focused on testing the HIV risk calculator and assessing its relevance and appropriateness of the questions among young people including young key population. |
| The discussion lacks critical reflection on how the findings relate to the available literature | The discussion is updated. |
| and, although, young people were involved in designing the app, it’s not clear whether young people were involved prior to this – ie. In deciding that an app would be most likely to address their barriers to HIV prevention services. | Young people were only involved from the design and development of the HIV risk calculator. However, based on literature review on social desirability bias on HIV prevention studies and anecdotal data from the researcher’s involvement in clinical trial research, it seems that there is a need for an anonymised and confidential self-assessment tool for HIV risk among young people. As a researcher in a HIV research filed and working in behavioural clinical trial, I identified that young people had a tendency to over report or under report HIV risk hence I had an inspiration to come up with a self-assessment HIV risk tool that has the potential to reduce under reporting or over reporting HIV risk activities. |
| It is unclear from the title, abstract and introduction that the study includes young key populations. Key populations are briefly mentioned in the abstract but then not in the introduction or under recruitment of PGD participants. It seems the focus was young people and young key populations – this should be made clearer in the title, abstract and introduction. | The title, abstract and introduction have been amended to show that the study included young key populations.  The following sections have been amended to reflect the change:  Title  The title has been changed from: “Developing a youth-friendly internet-enabled HIV risk calculator: A collaborative approach with young people aged 18–24 years living in Soweto, South Africa  to  “**Title:** Developing a youth-friendly internet-enabled HIV risk calculator: A collaborative approach with young people including key population aged 18–24 years living in Soweto, South Africa”.  Abstract: Background  Changed from “This paper describes the process of developing a youth-friendly internet-enabled HIV risk calculator (HRC) in collaboration with young people. “ to “This paper describes the process of developing a youth-friendly internet-enabled HIV risk calculator (HRC) in collaboration with young people including young people from the key population aged between the ages of 18 - 24.” |
| Related to this, the PGD included young people and asked them to comment on the questions included in the risk assessment. The IDI focused on key population’s preference for an app vs internet-enabled risk assessment tool and its appearance. Why weren’t young people in general and young people that are members of a key population not asked both? This needs clarification, as features important to key populations may not be important to individuals who do not belong to a key population; similarly, key populations might have considered some questions to be missing, e.g. related to sexuality and gender expression to be lacking from the assessment tool. | The IDIs included young people and especially targeted to include key population. The PGDs recruited young people irrespective of gender, sexual identity and practices. Gay, lesbian, bisexual and MSM also were identified in the PGDs even though they were fewer than in the IDIs. The PGDs formed phase one of the study which focused on developing and designing the initial prototype of the HIV risk calculator. Phase one of the study also focused on reviewing the HIV and STI assessment questionnaire.  The IDIs focused on testing the HIV risk calculator and assessing for its relevance and appropriateness of the questions among young people including young key population. |
| Later in the manuscript it states that the risk assessment is for HIV as well as other STIs, this should be made clearer in the introduction and provide information on STI burden (other than HIV) among young people in South Africa. | The HIV risk calculator is for self-assessment of HIV risk and does include some questions about history of testing positive for STIs. One question is asked about STI: Have you at any time suffered from STI such as genital wards, genital herpes, chlamydia and gonorrhoea?  As such we have removed the statement that suggest that the HIV risk calculator is for HIV risk assessment and other STIs. |
| One limitation of the study is that, although the study involved youth in the development of an app, did the authors discuss with young people how best to address barriers to services? It would seem that an important first step would be to ask young people how to improve their access to HIV and STI testing and treatment/prevention services. What was the rationale for developing an HIV risk assessment tool? And how is it anticipated that such a tool could improve their access to HIV and STI services? | Young people in both the PGDs and IDIs were asked about challenges to HIV counselling services, however the data is not reported in this paper because the aim of this papers is to report on the process of developing the HIV risk calculator.  The rationale to develop an anonymised and confidential HIV risk assessment tool emerged from the researcher’s experience working with young people in HIV prevention clinical trials. The researcher identified that young people often underreported on the number of sexual partners and condom use and the type of partner/sexual relationships they engaged with during HIV counselling and testing. Young people who reported one sexual partner, consistent condom use and knowing their partner’s HIV status seroconverted during the course of the study. During post test counselling, young people who tested HIV positive would confess that they often lied about their sexual behaviours due to fear of being judged and being taken off the clinical trial if they reported not using condoms all the time. As an adherence coordinator I saw there was a need for an anonymous and confidential tool for self-assessment of HIV risk.  The HIV risk calculator generates an HIV report that profiles the HIV risk of the user depending on the responses that they give from the HIV risk questions. In addition to providing the HIV risk profile, the HIV risk report include information on where young people can access HIV counselling and testing services. |
| At times in the manuscript, there is mention of the tool being about HIV prevention; other times it states that the tool would help improve access to HIV testing, prevention and treatment. Relating to the point above this, it is not clear what the app is intending to achieve and how this tool expected to contribute to HIV prevention? This needs clarification by clarifying what information the tool is intended to give young | The HIV risk calculator aims to provide young people with a tool to self-assess HIV risk and encourage HIV testing. Assessing for HIV risk can encourage testing for HIV and consequently prevent HIV infections among young people. On completion of the HIV risk calculator, a sexual risk report is generated and provide information about the different sexual activities and behaviour that is considered high risk and why they are considered high risk. The report also provide details of where young people can test free for HIV and receive HIV counselling. |
| Overall the discussion needs to reflect more critically on the study findings, and how these relate the published literature. For example, have other studies explored young people’s understanding of questions included in sexual behaviour questionnaires? How have other studies engaged youth in the development of interventions for youth – there is a growing body of literature on youth engagement that would strengthen the discussion. What other studies have used mHealth to improve access to HIV/STI services among young people, and how? | The discussion is updates accordingly to reflect reviewer comments. |
| Many of the references included in the manuscript are quite old, related to the point above, there is a need to include more recent evidence in the paper. | The introduction of the manuscript has been updated with recent references. |
| Abstract:  Change HIV/AIDS to HIV epidemic | HIV/AIDS change to HIV epidemic on the background section of the abstract |
| Specify age when referring to young people, and include prevention here – as the following sentence is about barriers to accessing HIV prevention services | Age range added on last sentence of the background section of the abstract. The sentence now reads as follows “This paper describes the process of developing a youth-friendly internet-enabled HIV risk calculator (HRC) in collaboration with young people aged 18 – 24 years old. |
| The sentence “Barriers to confidentiality …” needs rephrasing. I think the sentence aims to say that confidentiality is a concern among young people and thus acts as a barrier to service access, and that online services could address this barrier – but it’s not clear if that’s correct from how the sentence is currently written | The sentence now reads as follows “Concerns on confidentiality impedes on the utilization of HIV prevention services which signals the need for HIV prevention measures that tap discretely into youth-friendly platforms.” See background section of the abstract. |
| “Using a qualitative research design..” remove the word design from here | The words “a” and “design” were removed, the first sentence of the methodology section and now reads as follows “Using qualitative research, we conducted a cross-sectional exploratory study with young people including young people from the key population” |
| It’s not clear what is meant by “we conducted a cross-sectional exploratory study of young key populations” consider rephrasing this | The first sentence of the methodology section has been rephrased and now reads as follows “Using qualitative research, we conducted a cross-sectional exploratory study with young people including young key population” |
| LGBT individuals among other populations are mentioned in the abstract – is the study focussed on young key populations? If so, this needs to be made clear earlier on in the abstract and in the title | The first sentence of the methodology section has been rephrased and now reads as follows:  “Using qualitative research, we conducted a cross-sectional exploratory study with young people including young key population”  and the title has been updated and now reads as follows:  “Developing a youth-friendly internet-enabled HIV risk calculator: A collaborative approach with young people including young key population aged 18–24 years living in Soweto, South Africa” |
| “…mobile phone usage questionnaire..” is not clear, this needs rephrasing | “…mobile phone usage questionnaire changed” to “questionnaire on mobile phone usage”, throughout the manuscript. |
| The abstract should include details on how many participants were included in the study | The number of participants has been added on the methodology section of the abstract and Table 1. |
| Author Summary  The sentence starting “Facilities…” needs some rephrasing | As suggested, the sentence is rephrased on the author summary and now reads as follows:  “Facilities are available and in place to prevent, test for and treat HIV” |
| Introduction  The introduction needs to focus more on the risk of HIV among young key populations, as the target population for this study; | The introduction has been revised to focus on the HIV-related risks among young key populations. Moreover, it highlighted their vulnerabilities to the epidemic and the barriers that impede their access to HIV-related support. The introduction also explored the relevance of mHealth as an innovative method that aligns with the study's objectives. (See *page 4 to 7*) |
| The sentence “Young South Africans are predominantly…” needs some rephrasing, not clear what is meant by this – perhaps that they are at increased risk of HIV? | The sentence “Young South Africans are predominantly at risk, which demands innovative and revolutionary HIV prevention methods and initiatives” changed to read as follows:  “Young South Africans are at increased risk of HIV, which demands innovative and revolutionary HIV prevention methods and initiatives”. See page 5 paragraph 2. |
| It’s not clear how an HIV risk assessment tool would address barriers to confidentiality (long queues, sharing facilities with adults etc) among young people – this needs clarification, i.e. how would a mobile-phone based risk assessment tool would improve service access without addressing the barriers highlighted? | The HIV risk assessment questionnaire was used to collect data on HIV risk among young people . The HIV risk calculator was not was not developed to address structural barriers to HIV prevention and management service accessibility. The HIV risk calculator was developed to address anonymity and confidentiality of information related to sexual activity, condom use and other HIV related questions. |
| The sentence “…while many app have been developed for prevention…” needs references and please include examples of available app | We have added examples on some of the apps that have been developed for HIV prevention and this is addressed in the introduction section, paragraph 6. |
| The last paragraph on page 5 is more appropriate for the methods | Paragraph moved to methods section. See page 15, paragraph 2. |
| The final paragraph should describe the aim of the study, ie. The second paragraph of page 6 could be rephrased into the aim and added to the end of the last paragraph of the introduction  Methods | The second paragraph of page 6 was rephrased and moved to the last paragraph of the introduction. See page 8, last paragraph. |
| Spell out the acronyms included in the methods (e.g. PGD) | Acronyms included in the methods section are written in full. PGD changed to peer group discussion; IDI changed to in-depth interview and ACAB changed to adolescent community advisory board. See page 9-10. |
| Remove the term cross sectional – this is reserved for quantitative studies | The term cross sectional is removed on page 2, first sentence of the first paragraph under the section Methodology and again on page 9 under the section study design. |
| Have the first paragraph be study population and location, then the second is study design | The first paragraph under the methods section has changed to study population and the second to study design as suggested. See page 9 |
| Remove the word “massive”, rather give an indication of how many patients the walk-in clinic sees daily (for example) | The word “massive” is removed and the sentence now read as follows:  “The unit has a walk-in HIV counseling and testing (HCT) service facility, accessible to anyone wanting to test for HIV.” See page 9 |
| The acronym HCT is not commonly used anymore, revise to HIV testing services (HTS) | The acronym HCT is changed to HTS as suggested. See page 9 under study population and setting. |
| The first sentence under Recruitment process needs some rephrasing – move participated in the study before breaking down participation by each data collection method | Sentence corrected and moved up to the last sentence under study population and setting. See page 9. |
| Under the IDI participants heading, avoid the terms: “hard-to-reach” instead use marginalized, hidden, or minoritized; avoid “subjects”, instead use individuals and/or study participants; instead of access consider reach lesbian, gay etc | The terms “hard-to-reach” and “subjects” were replaced with the terms “marginalised” and “individuals”, respectively. See page 10, second sentence under the section on IDI Participants. |
| It says here young people were asked to recruit other people who met the eligibility criteria – but these should be included under study population as it’s not clear until this section that young key populations are among the study population | We have moved this information to the section on study population as suggested. See page 9 |
| On page 8, under peer group discussions, it’s not clear what was included in the 10 minute risk assessment questionnaire; also it is not clear what “assessing for other STIs..” means? Perhaps self-reported ever having had an STI? This needs clarification, also which STIs? Or were they asked about any STI (other than HIV) | For details on HIV and STI risk assessment questionnaire, see section on data collection, HIV and STI risk assessment questionnaire on page 14 paragraph 1.  The sentence “…followed by a 10-minute paper-based HIV risk assessment questionnaire which included assessing for other Sexually Transmitted Infections (STIs)” was changed to read as follows:  ***“…***followed by a 10-minute paper-based HIV and STI risk assessment questionnaire.” See page 11 under data collection procedures: Peer group discussion.  In addition, the following sentence was added to clarify what was assessed for STIs.  “To assess the history of STIs other than HIV, the questionnaire asked the following question: have you ever tested positive for STIs such as gonorrhoea, syphilis, Herpes.” See page 14, paragraph 1. |
| Page 9, first paragraph – this is the first time it is stated that the risk assessment will also assess for risk of STIs other than HIV, this should be made clearer in the introduction (i.e. that the risk assessments is for STIs, including HIV) | The HIV risk calculator if for HIV risk but included a question on the history of testing positive for other STIs other than HIV. The statement has been removed. |
| In this same paragraph, why were participants asked to complete this as a group? | Participants were not asked to complete the survey in group, however participants discussed the first five questions in a group setting, to assess the relevance and comprehensibility of the questions in the HIV risk calculator. See page 12, paragraph 2. |
| The labelling on figure 1 (and later figures) need to be clearer (larger font, no acronyms etc) | The labelling on all figures have been enlarged for visibility and acronyms removed. |
| Under in-depth interviews, the term once-off is not necessary here | The term “once-off” is removed. See page 12, first sentence under the section on In-depth interviews. |
| Page 10, you can remove the text in brackets reminding the reader who the key populations are | The text in brackets is removed. See page 13, paragraph 1. |
| Considering this is a qualitative study, the measurement section is not necessary particularly as this section is not about measurement but about topics explored. Rather this information could be integrated in the data collection section | The term “measurements” is removed as suggested. The section is on data collection procedures. |
| The data analysis section does not need two headings (ie. Don’t need data analysis and qualitative data analysis) | The second heading reading “qualitative data analysis” is removed. See page 15. |
| It says here: “All codes were discussed, and any discrepancies were resolved.” But earlier it states that only one person coded the data, what discrepancies are meant in this sentence? | The first four transcripts (2 PGDs and 2 IDIs) were coded by the lead researcher (first author) and the MA student (second author). Hence the reference to “main coder”.  The statements were corrected to read as followings:  The first two PGD and two IDI transcripts were read and coded by the first and second author based on the initial framework, while allowing identification of emerging codes. The remaining transcripts were coded by the lead researcher (first author) following a line-by-line technique to capture codes that would otherwise emerge from data that was not initially part of the pre-existing codes.  “Final codes were discussed and approved by the supervising researchers.” See page 15-16 |
| Consider revising the sentence: “Descriptive statistics and frequencies were used to analyze socio-demographic characteristics and mobile phone usage data…” to Socio-demographic and mobile phone usage data were described. … | The sentence “Descriptive statistics and frequencies were used to analyze socio-demographic characteristics and mobile phone usage data…” is changed and now reads as follows:  “Socio-demographic characteristics and mobile phone usage data were described using SPSS Version 25.0.” See page 16, paragraph 2. |
| The first paragraph mentions that 2 individuals were transgender – were they transmen or transwomen or both? | The term “transgender” is changed to transwomen to indicate that 2 individuals were transgender men to women. See page 16, first sentence under section on participants demographics and mobile phone usage under results section. |
| The last sentence of the first paragraph states: Overall, 86% (30/35) had access to the internet via a mobile phone, tablet, laptop, or computer. By saying overall the reader assumes among all study participants – the denominator should be the 40 individuals, with the % changed accordingly. | Yes, all study participants had to answer the question on internet access. The correction is made, and the sentence changed from:  “Overall, 86% (30/35) had access to the internet via a mobile phone, tablet, laptop, or computer.” To the following statement:  “Overall, 72.5% (29/40) had access to the internet via a mobile phone, tablet, laptop, or computer.” |
| Although quotes are included in the table, there should be additional quotes included in the text to support the assertions made in the text – for the PGD and the IDI | Additional quotes were included in the text to support the assertions made in the text. See section on paragraphs headed: Feedback on the risk assessment for HIV and STI questionnaire. And section headed: Feedback on developing a youth-friendly internet-enabled HRC |
| Statements such as: All questions considered problematic were revised in collaboration with participants and discussed further with the research team for corroboration. Questions approved by participants and the research team were then sent to the software developer to incorporate into the HRC. Should be moved to the discussion – the results are to present study findings only. | The statements were moved to the discussion section. See page 31, paragraph 2. |
| The first paragraph of the discussion needs to restate the key findings of the study | The discussion is updated and restate the key findings of the study. |
| In the second paragraph of the discussion, are there any studies that explored young people’s understanding of questions included in survey questionnaires? | To the authors knowledge, there are no studies that explore young people’s understanding of the questions included in the HIV and STI risk assessment question, particularly in South Africa. This statement is added in the discussion section on page 31 of the discussion section, first sentence in the third paragraph. |
| This paragraph states that the study expands on the limited evidence of the impact of mobile phones on risk assessment but you study does explore impact, rather it engages young people in the design of the app | We agree with the reviewer and therefore revised the sentence to read as follows:  This study adds to the limited existing literature on mobile phones on HIV risk assessment and behavioral data collection among young people in South Africa”. See page 31 of the discussion section last sentence of paragraph 3. |
| In the conclusion, it would be useful to state whether the tool will be evaluated and, if so, how? | The HIV risk calculator was evaluated and the following statement has added in the conclusion section:  The HIV risk calculator was evaluated as part of a PhD degree and results are available in the form of a thesis on the database of the University of the Witwatersrand in Johannesburg South Africa.  See page 35, last sentence of the conclusion section. |

| REVIEWER2 |  |
| --- | --- |
| Comments | Response |
| Reviewer #2: This manuscript focuses on an important health disparity and makes an important contribution to the literature by documenting the process used to engage young people in the development of an mHealth health promotion tool. I have a few suggestions, mainly to enhance the clarity of the information presented. | |
| 1) Populations of focus: The Abstract and Author Summary mention persons who inject drugs as a population of focus for this study (along with LGBT individuals, MSM, and female sex workers); however, the Methods and Results in the main text do not describe the specific recruitment of persons who inject drugs for the study. | Thank you for this important observation. Persons who inject drugs were not included in the sample of this study. Persons who inject drugs were removed from the manuscript. See abstract and authors summary for changes. |
| 2) In addition, in some places LGBT individuals and MSM are presented as separate populations and in others (e.g., the subsection titled "Semi-structured interview guides") it seems that these two populations may have been combined into a single LGBT category. These aspects could be made clearer/more consistent. | LGBT individuals and MSM are presented separately in this study. Therefore, we have made changes to present LGBT individuals and MSM separately throughout the study. See subsection titled "Semi-structured interview guides", last paragraph of page 14 and first paragraph of page 15. |
| 3) Data collection: It would be helpful if the authors could explain in the Methods section the languages used in the discussions/interviews vs. the written assessment. This is explained later (e.g., in the Limitations section), but it would be clearer if stated more explicitly when first describing data collection. | The following changes were made to explain languages used in the interviews and discussions. For PGDs the sentence was added stating that “All PGDs were conducted using participant’s preferred language which were IsiZulu, Sesotho and English.” See page 11, fourth sentence of paragraph under Peer group discussions.  And for IDIs the following statement was added:  “IDIs were conducted in the participants’ preferred language which were IsiZulu, Sesotho and English.” See page 13, third sentence of the paragraph under In-depth interviews section. |
| 4) In addition, were both the demographic and mobile phone usage questionnaire and the HIV risk assessment questionnaire paper-based? As written, the risk assessment questionnaire is described as paper-based but the format for the demographic/mobile phone usage questionnaire is not specified. | The demographic and mobile phone usage questionnaire and the HIV risk assessment questionnaire were paper-based. The following statement is added:  “Participants completed a paper-based self-administered, five-minute demographics questionnaire and a questionnaire on mobile phone usage followed by a 10-minute paper-based HIV and STI risk assessment questionnaire.” See page 11, paragraph 2 and page 13, first sentence of the first paragraph. |
| 5) Demographics: Given that sex workers were one of the populations specifically recruited for the study, and they are a population traditionally considered difficult to reach by researchers, it would be valuable, if possible, if the authors could include in the demographics section the number of participants who self-identified as sex workers. | The following statement has been added to the participants demographics section of the results:  “Two participants identified as female sex workers.” See page 17, first paragraph. |
| 6) Assessment development process: I was left with two main questions about the process used to develop the HIV risk assessment tool. First, regarding the question on the assessment about race and participants' concerns about it further stigmatizing specific groups: the authors state that the decision was made to leave this question on the assessment and to provide an explanation about its purpose to the young people. Was this explanation just provided to PDG participants, or was an explanation embedded in the assessment for future users to read when they get to that question? Second, more information about the transition to a chatbot format would be helpful. Is the chatbot an app (vs. a website), and/or how does it address the concerns participants brought up about data limitations? | Participant in the PGD were given an explanation on why the question on race was not taken out of the HIV risk assessment questionnaire. This explanation was not embedded in the assessment for future users to read when they get to the question. The authors agree that this explanation will be embedded in the updated version of the HIV risk calculator.  Description of the chatbot format documented on page 15 and reads as follows:    “…The youth-friendly internet enabled HRC is an internet enabled webpage with 30 self-reported item questions about HIV risk behavior.”  Due to limitations on funding, an app could not be developed which address concerns of data limitation as data/wifi would only be required for downloading the App and not navigating the app. |
| 7) Tables: Some of the abbreviations in the Tables (e.g., IDIX and IDI WOW) were not clear to me. It would be helpful if these could be defined. In addition, in some cases the participant age is included with the quote, in others the date, and in others neither. I recommend making this format more consistent, if possible. | The format for all extract has been made uniform and updated. The referencing of all extract is as follows: for IDIs: “***IDI, gender, age*** *and for PGDs:*  ***PGD ACAB, mix gender and age range*** *or* ***PGD, gender and age range”.*** |
| 8) Terminology: For consistency throughout the manuscript and to use the most up-to-date language, in the subsection titled "Semi-structured interview guides" I suggest changing "LGBTs" to "LGBT individuals", and changing "homosexual" in Results to "lesbian or gay". | Homosexual was changed to ‘lesbian or gay’ see page 17 first paragraph of the results. LGBTs was changed to LGBT individuals see page 15 under subsection titled "Semi-structured interview guides". |
| Again, both the development process and intervention presented in this manuscript are innovative and of interest to those both in research and practice. My main suggestion is to clarify the points above. | |

ADDITIONAL CHANGES

We have added participants demographics table.
