# Supplementary material for: Developing a youth-friendly internet-enabled HIV risk calculator: A collaborative approach with young key populations, living in Soweto, South Africa
Source: PLOS Digit Health. 2025 Jan 31;4(1):e0000672. doi: 10.1371/journal.pdig.0000672 (PMC11785273; doi:10.1371/journal.pdig.0000672)
Supplement: Attachment — Submitted filename: Reviev comments.docx [file pdig.0000672.s005.docx]

The research article, "Developing a youth-friendly internet-enabled HIV risk calculator: A collaborative approach with young people including key population aged 18–24 years living in Soweto, South Africa," focuses on creating an HIV risk tool for youth.

It conducts a qualitative study with young people in Soweto, South Africa, aiming to create an accessible and interactive tool.

The manuscript is well-structured, addressing a crucial health issue, but it needs more precise language in methodology and results. It should explicitly discuss how it fills literature gaps, particularly in mHealth for HIV prevention.

The paper commendably engages with youth but should delve deeper into the tool's real-world impact, addressing implementation challenges. While it presents data clearly, incorporating more visual aids could enhance understanding. Ethical considerations are well-handled, yet further discussion on the implications of involving young populations would strengthen it.

The manuscript briefly mentions future research but could expand on this, discussing potential scalability.

Minor revisions could significantly enhance its clarity and expand its impact, making it a valuable field contribution.
