# Supplementary material for: Developing a youth-friendly internet-enabled HIV risk calculator: A collaborative approach with young key populations, living in Soweto, South Africa
Source: PLOS Digit Health. 2025 Jan 31;4(1):e0000672. doi: 10.1371/journal.pdig.0000672 (PMC11785273; doi:10.1371/journal.pdig.0000672)
Supplement: Attachment — Submitted filename: RebuttalLetter-PDIG-D-23-00013-R3-30-MAY-2024.doc [file pdig.0000672.s007.doc]

**Rebuttal letter for manuscript**: PDIG-D-23-00013R3

**Title**: Developing a youth-friendly internet-enabled HIV risk calculator: A collaborative approach with young people including key population aged 18–24 years living in Soweto, South Africa”.

Dear Editor

Thank you very much for the manuscript review and for affording us the opportunity to respond to journal and reviewer requirements and queries, which we do so below in blue text.

**Response to reviewer # 3**

Comment 1: The manuscript is well-structured, addressing a crucial health issue, but it needs more precise language in methodology and results. It should explicitly discuss how it fills literature gaps, particularly in mHealth for HIV prevention.

**Response 1: The literature review in this manuscript highlight the importance of leveraging platforms that young people are familiar with and responsive to. As such it posits that mHealth interventions for HIV prevention will provide an opportunity for innovative ways of preventing HIV risk amongst young people. The literature also affirms that there are many existing HIV prevention apps/tools for young people, however these existing tool are not made use of due to lack of awareness and lack of marketing. Sometimes because the targeted user is not part of the development of the apps from the start.**

Comment 2: The paper commendably engages with youth but should delve deeper into the tool's real-world impact, addressing implementation challenges.

**Response 2: The focus of this manuscript is not on implementation, but the process engaged in developing an HIV risk assessment tool in collaboration with young people in Soweto, South Africa. Implementation challenges will be addressed in future manuscripts from the data available.**

Comment 3: While it presents data clearly, incorporating more visual aids could enhance understanding.

**Response 3: Thank you. The supplementary documents present the HRC from a prototype to the different personas young people suggested for aesthetic purposes of the HRC, so that it does not look like an HIV related tool/app.**

Comment 4: Ethical considerations are well-handled, yet further discussion on the implications of involving young populations would strengthen it.

**Response 4: The ethical considerations for including young people in HIV related research were addressed in the section on ethical considerations. Involving young people that are considered vulnerable to HIV and other sexually transmitted infections was a commendable step taken by the research team to include young people considered key population and access to these young people was achieved through youth projects that focused on young people and care was taken to ensure their confidentiality by making sure that young people that identified as LGBT and sex work were only involved in in-depth interviews and no identifying information was collected as part of the research.**

Comment 5: The manuscript briefly mentions future research but could expand on this, discussing potential scalability.

**Response 5: The HRC will be tested for usability and acceptability with different populations in different contexts in Sexual and Reprodctive services. The plan is to include the instrument within existing Sexual and Reproductive services to make the tool visible to the intended target. Ample tools on Sexual and Reproductive services and HIV prevention exist, but the targeted audience is not aware of them thus not using them.**

**Response to reviewer # 4**

**Title**

Comment 1: The title is a little confusing/non-specific as to who the key population is.– it is young people aged 18-24 or young people aged 18-24 who are also members of the group at high risk of infection – LGBT, MSM and female sex workers? It should to be clearer who the key populations are.

**Response 1:The title is revised and now reads as follows: Developing a youth-friendly internet-enabled HIV risk calculator: A collaborative approach with young key population, aged 18–24 years living in Soweto, South Africa**

**Abstract**

Comment 2: In the abstract, qualitative analysis was conducted in Nvivo (software) yet in the methods a manual process is described with no mention of Nvivo. Can the author clarify and correct as necessary.

**Response 2: The initial process of data analysis involved the development of a coding framework which was conducted manually by listing apriori codes/deductive codes based on the literature review and the interview guide. Once the coding framework was created, authors uploaded the initial codes into Nvivo and continued to code the transcripts allowing new codes to emerge. A clarification is added under data analysis in the methods.**

Comment 3: The term baseline data is a little confusing as it relates to the HIV risk assessment tool– it could be misinterpreted as baseline risk assessment data rather than perceptions of the tool to calculate HIV risk in the pre-development stage of the app. This should be made clearer.

**Response 3: The term baseline is removed from the statement as it basically referred to data collected on demographics and HIV risk assessment questionnaire.**

Comment 4: ‘iterative’ is referred to in the abstract and main text conclusion – is this supposed to be ‘interactive’ as iterative is not mentioned elsewhere?

**Response 4:**

**Yes, we have replaced the term ‘iterative’ with ‘interactive’. As part of the findindings, particiants shared that they want the HRC to be interactive whereby they are able to ask questions and get responses on the app/web tool.**

**Main text**

Comment 5: Some abbreviations are not defined at first instance or at all while others are defined multiple times or defined but not used again. HRC is not defined at its first use in the main text and not until the methods. LGBT and MSM are defined more than once – in intro and methods. FSW is defined but used only once in the main text, similarly for NICD. HCT is still used in the methods and tables but has been corrected elsewhere to HTS as per the introduction. It also still appears in some tables. The general rule of using abbreviations only if the term is used 3 or more times should be applied and a list of abbreviations included in the supplemental information or end of manuscript and any table abbreviations included in the table legends.

**Response 5: HRC written in full at first use in the abstract. See pg xx under background in the abstract section. We removed the following abbreviations: FWS, NICD and HCT is corrected to HST throughout the manuscript.**

**Methods**

Comment 6: The phrase “In 2017, HIV prevalence in the general population (15–49 years) in Gauteng sitting at the was estimated at 17.6% [30].” Needs rephrasing – sitting or setting? at the…

**Response 6: the phrase was corrected by removing the words ‘sitting at the”. It now reads as follows: “In 2017, HIV prevalence in the general population (15–49 years) in Gauteng was estimated at 17.6%”**

Comment 7: Clarification is needed on where the participants for the IDI’s were recruited. Later it states that “In addition, clinic-based recruitment through PHRU’s HCT facility and other ongoing projects was used for recruiting young key populations” – in addition to what? Where was the purposive sampling applied, who made the initial identification of potential participants to begin the chain referral process?

**Response 7:** **The young people who were sexually active were recruited via active community recruitment where study fieldworkers went into the community to hand out pamphlets and where possible take contact details (cell phone number, age, gender and residence area) of individuals who showed interest to participate in the study. Following a completed IDI, participants were asked to refer other young people they know who might meet the criteria for participation.**

Comment 8: The research team and researcher reflexivity should be clarified in the methods as per the CONSORT and SRQR guidelines for qualitative research on the EQUATOR site. <https://www.equatornetwork.org/?post_type=eq_guidelines&eq_guidelines_study_design=qualitativeresearch&eq_guidelines_clinical_specialty=0&eq_guidelines_report_section=0&s=>

**As part of reflexivity, the researcher MM, shared her goals and her interest in the research on HIV prevention for young people. As te researcher, MM was a PhD student during data collection and has had extensive research experience in qualitative data collection and analysis. The research assistants who were involved in data collection were both MA students and received training in qualitative research methods. The researcher and research assistants had no prior knowledge or interaction with the particiants. A rapport was built through initial introduction to the participants prior the PGDs and in-depth interviews. Female partipants were interviewed by the female research assistant and the male participants were matched with the male interviewer, whenever possible.**

**MM’s initial assumptions were that young people are not aware of their risk for HIV and other sexually transmitted infections. Through the discussion with the particiants, it became a reality that young people cannot define what HIV risk is and did not understand the importance of completing an HIV risk assessment questionnaire during HIV counseling and testing. This realization may have biased the data analysis hence the data analysis involved 2 researchers and also went through the research team for approval of codes and themes generated during the analysis.**

**Response 8:**

Comment 9: I couldn’t tell from the figures, if the text in the prototype was clearly visible

**Response 9: The text on the figures are enlarged. See supplementary documents**

Comment 10: The line ‘After completing the HRC prototype’ needs clarification – did they complete the risk assessment using the prototype (the HRC) or is this referring to the discussion of the prototype?

**Response 10: The HIV risk assessment question was paper based as specified under data collection. On day 2 of the PGDs, participants were presented with a basic HRC prototype as stated in the last paragraph of page 9 and first paragraph of page 10. “Participants were first reminded of the intention to develop a youth-friendly internet-enabled HRC and were shown an early-stage prototype (Figure 1)—comprising five questions relating to age, gender, race, HIV status, and partner’s HIV status—via an online link on a tablet. Finally, participants discussed the first five questions in a group setting to assess the relevance and comprehensibility of the questions on the HIV Risk calculator.”**

Comment 11: It would be helpful and in line with standards for reporting of qualitative research to provide access to the discussion/interview guides. These are also the basis on which the initial coding framework for the data analysis was developed so it would improve transparency if they are made available.

**Response 11: The interview guide for PGDs and IDIs are shared as supplement 4 and 5, respectively.**

Comment 12. From the methods the ACAB discussion involved a different iteration of the prototype design to the PGDs and IDIs. It might help the reader to provide a schematic of the timeline for the PDGs, IDIs and ACAB, what activities where undertaken by which group (where quantitative and qualitative data derives from) and where there was iteration in the prototype design.

**Response 12: See a schematic representation of the data collection, supplementary 3.**

Comment 13: In the data analysis it needs to be clearer as to whether the outputs from the 3 qualitative data collection activities were analysed together. It is a little confusing that in table 2 PGDACAB mixed gender participant commented on the wording and then the question was revised as per column 4, as from the methods, it reads that revisions were made following the suggestions from the first PGDs and IDIs, with the prototype being revised before the ACAB discussions

**Response 13: Discussions were conducted with participants through PGDs and later with IDIs. Feedback was shared with the software developers to implement suggestions for improvements. Discussion with ACAB also involved evaluation of the HIV risk assessment questions for comprehension and relevance for young people.**

**Results**

Comment 14: There is a duplication of one row in Table 1 – Highest level of education, Completed grade 12. The duplicate line also has an error in the percentage

**Response 14: The duplicated line is removed. See table 1**

Comment 15: I suggest defining what is meant by Pay Airtime - is this a local term? I am not familiar with it. It can be defined in the table legend.

**Response 15: Pay Airtime is taken from SAS variable names derived from the question: “how do you pay for airtime?”. We shortened it to ‘Pay Airtime’. We provide an explanation below the tale and mark it with an asterisk for the reader to pay attention**

Comment 16: In Table 2 the formatting with alternating white and the pale grey is a little confusing. “2) Difficult wording in questions” is grey as is the first row associated with it, but not the second row (white) This second row looks like it could be associated with (3). Some reformatting is needed to make this clearer and should be made consistent across all tables.

**Response 16: Table 2 and 3 are formatted by removing the grey highlight.**

Comment 17: The labelling for the quotes needs to be checked and corrected for consistency – e.g. IDI013; IDI-004; IDI 006; IDI,X; and IDI-016WOW. There seems to be multiple variations throughout the manuscript

**Response 17: The labelling for the quotes were checked and corrected for consistency – e.g. IDI013 changed to IDI-013; IDI-004 is correct; IDI 006 changed to IDI-006; IDI, X corrected to PGD, females, 18 – 24 years old; and IDI-016WOW corrected to IDI-016.**

Comment 18: Under the theme “Feedback on developing a youth-friendly internet-enabled HRC” table 2 is referred to. I think this is supposed to be table 3.

**Response 18: Yes, it is an error. Table 2 corrected to table 3. See pg xx under the theme “Feedback on developing a youth-friendly internet-enabled HRC”**

**Conclusion**

Comment 19: The first conclusion is not justified – “This study explored how the development of a youth-friendly internet-enabled HIV risk calculator could help young people to overcome the hurdles they face in accessing HIV testing, prevention, and treatment”. In line with study aim, it instead describes the process for developing the app using a qualitative and collaborative approach and identified areas for app improvement and modifications to potentially increase acceptability and correct use by the target population. However it doesn’t explore how the app can help the target population overcome the hurdles to accessing testing, prevention and treatment – I understand this is a goal of the app, but it is not explored here in my opinion through the resulting themes identified. Perhaps this conclusion is more appropriate to the app evaluation referred to.

**Response 19: We agree with the reviewer. The conclusion stating that This study explored how the development of a youth-friendly internet-enabled HIV risk calculator could help young people to overcome the hurdles they face in accessing HIV testing, prevention, and treatment”, has been removed.**

Regards,

**Dr Mamakiri Mulaudzi**

[**https://orcid.org/0000-0001-8495-2209**](https://orcid.org/0000-0001-8495-2209)

[**tel:+27119899831**](tel:+27119899831)**/011 717 8331**

[**https://assureafrica.com/**](https://assureafrica.com/)

[**mamakirim@assureafrica.com**](mailto:mamakirim@assureafrica.com) **/** [**mamakiri.mulaudzi@wits.ac.za**](mailto:mamakiri.mulaudzi@wits.ac.za)
