# Supplementary material for: Developing a youth-friendly internet-enabled HIV risk calculator: A collaborative approach with young key populations, living in Soweto, South Africa
Source: PLOS Digit Health. 2025 Jan 31;4(1):e0000672. doi: 10.1371/journal.pdig.0000672 (PMC11785273; doi:10.1371/journal.pdig.0000672)
Supplement: Attachment — Submitted filename: Reviewer comments.pdf [file pdig.0000672.s006.pdf]

Summary of research and overall impression

Thank you to authors for the opportunity to read this very interesting manuscript. This research describes a participatory approach to the development of an app targeted at young people for the self assessment of HIV risk, and to promote information on safe sexual practices and access to HIV testing services. Critically, it included potential users from marginalised groups at high risk of infection – namely LGBT people, men who have sex with men and female sex workers. It is an important piece of research as it identifies key challenges for young people in existing HIV risk assessment tools, namely the language used, understanding of questions and differing perceptions of the HIV risk assessment generally including specific variables such as race. The study identifies existing gaps in knowledge that future youth specific apps need to fill to ensure correct use, accurate assessment of risk and provision of understandable and engaging evidence based information and advice. Additionally, the study highlighted that confidentiality and discreteness were critical app features for potential future users and yet they still wanted a colourful and interactive interface to encourage use. The research approach is valid and the findings are well presented. The discussion discusses some of the interesting findings. Most conclusions are justified but the first listed (primary?) conclusion in the main text does not seem to be supported by the study design or findings. The study will be of interest not only to the HIV research community but also to those designing applications targeting young cohorts where the focus is around a heath issue that is subject to stigmatisation – obesity, violence against women and girls, mental health etc thus where users perceive a high need for privacy. From previous reviewers comments I can see the authors have made significant efforts to clarify and improve the manuscript however some minor issues remain outstanding that make the manuscript confusing or hard to follow at times and some typographical and formatting issues reduce the quality of the writing.

Specific areas for improvement

Title:

1. The title is a little confusing/non-specific as to who the key population is.– it it young people aged 18-24 or young people aged 18-24 who are also members of the group at high risk of infection – LGBT, MSM and female sex workers? It should to be clearer who the key populations are.

Abstract

2. In the abstract, qualitative analysis was conducted in Nvivo (software) yet in the methods a manual process is described with no mention of Nvivo. Can the author clarify and correct as necessary.
3. The term basline data is a little confusing as it relates to the HIV risk assessment tool– it could be misinterpreted as baseline risk assessment data rather than perceptions of the tool to calculate HIV risk in the pre-development stage of the app. This should be made clearer.
4. ‘iterative’ is referred to in the abstract and main text conclusion – is this supposed to be ‘interactive’ as iterative is not mentioned elsewhere?

Main text

5. Some abbreviations are not defined at first instance or at all while others are defined multiple times or defined but not used again. HRC is not defined at its first use in the main text and not until the methods. LGBT and MSM are defined more than once – in intro and

methods. FSW is defined but used only once in the main text, similarly for NICD. HCT is still used in the methods and tables but has been corrected elsewhere to HTS as per the introduction. It also still appears in some tables. The general rule of using abbreviations only if the term is used 3 or more times should be applied and a list of abbreviations included in the supplemental information or end of manuscript and any table abbreviations included in the table legends.

Methods

6. The phrase "In 2017, HIV prevalence in the general population (15–49 years) in Gauteng sitting at the was estimated at 17.6% [30]." Needs rephrasing – sitting or setting? at the…?

7. Clarification is needed on where the participants for the IDI's were recruited. Later it states that "In addition, clinic-based recruitment through PHRU's HCT facility and other ongoing projects was used for recruiting young key populations" – in addition to what? Where was the purposive sampling applied, who made the initial identification of potential participants to begin the chain referral process?

8. The research team and researcher reflexivity should be clarified in the methods as per the CONSORT and SRQR guidelines for qualitative research on the EQUATOR site. https://www.equator-network.org/?post_type=eq_guidelines&eq_guidelines_study_design=qualitative-research&eq_guidelines_clinical_specialty=0&eq_guidelines_report_section=0&s=

9. I couldn't tell from the figures, if the text in the prototype was clearly visible

10. The line 'After completing the HRC prototype' needs clarification – did they complete the risk assessment using the prototype (the HRC) or is this referring to the discussion of the prototype?

11. It would be helpful and in line with standards for reporting of qualitative research to provide access to the discussion/interview guides. These are also the basis on which the initial coding framework for the data analysis was developed so it would improve transparency if they are made available.

12. From the methods the ACAB discussion involved a different iteration of the prototype design to the PGDs and IDIs. It might help the reader to provide a schematic of the timeline for the PDGs, IDIs and ACAB, what activities where undertaken by which group (where quantitative and qualitative data derives from) and where there was iteration in the prototype design.

13. In the data analysis it needs to be clearer as to whether the outputs from the 3 qualitative data collection activities were analysed together. It is a little confusing that in table 2 PGD-ACAB mixed gender participant commented on the wording and then the question was revised as per column 4, as from the methods, it reads that revisions were made following the suggestions from the first PGDs and IDIs, with the prototype being revised before the ACAB discussions.

Results

14. There is a duplication of one row in Table 1 – Highest level of education, Completed grade 12. The duplicate line also has an error in the percentage.

15. I suggest defining what is meant by Pay Airtime - is this a local term? I am not familiar with it. It can be defined in the table legend.

16. In Table 2 the formatting with alternating white and the pale grey is a little confusing. "2) Difficult wording in questions" is grey as is the first row associated with it, but not the second row (white) This second row looks like it could be associated with (3). Some reformatting is needed to make this clearer and should be made consistent across all tables.

17. The labelling for the quotes needs to be checked and corrected for consistency – e.g. IDI013; IDI-004; IDI 006; IDI,X; and IDI-016WOW. There seems to be multiple variations throughout the manuscript.

18. Under the theme "Feedback on developing a youth-friendly internet-enabled HRC" table 2 is referred to. I think this is supposed to be table 3.

Conclusion

19. The first conclusion is not justified – "This study explored how the development of a youth-friendly internet-enabled HIV risk calculator could help young people to overcome the hurdles they face in accessing HIV testing, prevention, and treatment". In line with study aim, it instead describes the process for developing the app using a qualitative and collaborative approach and identified areas for app improvement and modifications to potentially increase acceptability and correct use by the target population. However it doesn't explore how the app can help the target population overcome the hurdles to accessing testing, prevention and treatment – I understand this is a goal of the app, but it is not explored here in my opinion through the resulting themes identified. Perhaps this conclusion is more appropriate to the app evaluation referred to.
